# Analysis of the relationship among land surface temperature (LST), land use land cover (LULC), and normalized difference vegetation index (NDVI) with topographic elements in the lower Himalayan region

**DOI:** 10.1016/j.heliyon.2023.e13322

**Published:** 2023-02-03

**Authors:** Waheed Ullah, Khalid Ahmad, Siddique Ullah, Adnan Ahmad Tahir, Muhammad Faisal Javed, Abdul Nazir, Arshad Mehmood Abbasi, Mubashir Aziz, Abdullah Mohamed

**Affiliations:** a Department of Environmental Sciences, COMSATS University Islamabad, Abbottabad Campus, 22060, Pakistan; b Department of Civil Engineering, COMSATS University Islamabad, Abbottabad Campus, Tobe Camp University Road Abbottabad 22060, Pakistan; c Department of Civil and Environmental Engineering, King Fahd University of Petroleum & Minerals, Dhahran 31261, Saudi Arabia; d Interdisciplinary Research Center for Construction and Building Materials, King Fahd, University of Petroleum and Minerals, Dhahran 31261, Saudi Arabia; e Research Centre, Future University in Egypt, New Cairo 11835, Egypt

**Keywords:** Land surface temperature, Land use land cover changes, Maximum likelihood classification, Normalized difference vegetation index

## Abstract

Land Surface Temperature (LST) affects exchange of energy between earth surface and atmosphere which is important for studying environmental changes. However, research on the relationship between LST, Land Use Land Cover (LULC), and Normalized Difference Vegetation Index (NDVI) with topographic elements in the lower Himalayan region has not been done. Therefore, the present study explored the relationship between LST and NDVI, and LULC types with topographic elements in the lower Himalayan region of Pakistan. The study area was divided into North-South, West-East, North-West to South-East and North-East to South-East directions using ArcMap 3D analysis. The current study used Landsat 8 (OLI/TIRS) data from May 2021 for LULC and LST analysis in the study area. The LST data was obtained from the thermal band of Landsat 8 (TIRS), while the LULC of the study areas was classified using the Maximum Likelihood Classification (MLC) method utilizing Landsat 8 (OLI) data. TIRS collects data for two narrow spectral bands (B10 and B11) with spectral wavelength of 10.6 μm–12.51 μm in the thermal region formerly covered by one wide spectral band (B6) on Landsat 4–7. With 12-bit data products, TIRS data is available in radiometric, geometric, and terrain-corrected file format. The effect of elevation on LST was assessed using LST and elevation data obtained from the USGS website. The LST across LULC types with sunny and shady slopes was analyzed to assess the influence of slope directions. The relationship of LST with elevation and NDVI was examined using correlation analysis. The results indicated that LST decreased from North-South and South-East, while increasing from North-East and South-West directions. The correlation coefficient between LST and elevation was negative, with an R-value of −0.51. The NDVI findings with elevation showed that NDVI increases with an increase in elevation. Zonal analysis of LST for different LULC types showed that built-up and bare soil had the highest mean LST, which was 35.76 °C and 28.08 °C, respectively, followed by agriculture, vegetation, and water bodies. The mean LST difference between sunny and shady slopes was 1.02 °C. The correlation between NDVI and LST was negative for all LULC types except the water body. This study findings can be used to ensure sustainable urban development and minimize urban heat island effects by providing effective guidelines for urban planners, policymakers, and respective authorities in the Lower Himalayan region. The current thermal remote sensing findings can be used to model energy fluxes and surface processes in the study area.

## 1 Introduction

Land surface temperature is a significant parameter used to study surface energy balance, climate change, and various earth's physical and chemical processes [1,2]. In mountainous areas, the Spatio-temporal distribution of the LST is highly affected by topographical gradients, vegetation, and LULC types. Topography affects LST by changing surface illumination conditions under different slopes and aspects. For example, in the Northern hemisphere, the north-facing slopes experience less radiation than the southern ones and therefore have low LST. Absorbing and reflecting solar radiation energy and controlling latent and sensible heat exchange, vegetation can affect LST. NDVI is frequently used to investigate the LST relationship with vegetation [3]. LST is also affected by LULC types via surface reflectance and roughness, resulting in LST differences between LULC types [4,5]. Because a variety of factors influence the relationship between LST and NDVI such as urbanization, LULC changes, types of vegetation, soil, and water [6]. LULC change is an important factor affecting LST and NDVI relationship of a region [7]. For example [8] found that LST in the urban areas of the Lower Himalayan region has increased several folds due to LULC changes in the past 30 years. This was primarily due to impervious surfaces replacing vegetation [9]. This resulted in the formation of urban heat islands (UHI) [10]. Elevation [11], solar radiation level [12], water cooling effect [13], and soil moisture all influence the relationship between NDVI and LST [14]. The effects of topographic parameters (elevation, slope and aspects) were evaluated by Ref. [15] and found that LST decreased with an increase in elevation. They also noted LST variations with slope and aspects in the study area due to changes in the radiation conditions. A negative relationship of elevation and slope with LST while with aspect angle was reported by Ref. [16]. They also found low LST in the shady slope than on the sunny side. It has been concluded that elevation, slope, and aspect affected LST because of variations in solar radiation. On a small scale, slope and aspect directly affect potential radiation and thermal load.

The Himalayan range is over 250–300 km from Nanga Parbat (Pakistan) to the west (Namche Barwa) and east to the Tibet plateau. Based on elevation, the Himalayan range is classified into sub-Himalayan, Lower, and Greater Himalayan ranges. It is the main source of precipitation in Asia by intercepting summer monsoons coming from the Bay of Bengal and the Arabian Sea, which provides fresh water to the 1.3 billion population [17,18]. It also prevents the cold continental air of central Asia and forces the south-easterly rain-bearing wind to give up moisture content before crossing the range northward [19]. Due to complex topography, various soil types, and climate conditions, the Himalayan region is the hotspot of biodiversity and has more than 10,000 plants and more than 1200 species of fish and amphibians, reptiles, birds, and mammals [9]. Flora and fauna of the Himalayans are undergoing structural and compositional changes due to climatic variations resulting in various species shifting to higher elevations [20]. Human-induced LULC, LST, and vegetation changes globally affect the mountainous regions' biodiversity. This is particularly true for the Himalayan Mountain range, where increasing human activities cause LULC and LST changes, affecting the region's overall climatic regime and biodiversity [21].

The present study focuses on the Northern KP, located in a lower Himalayan region. The study area is one of Pakistan's best-known tourist areas and has a rich biodiversity. Most of the previous LST studies were conducted in urban environments using MODIS and Landsat data. For example [22], investigated LST dynamics in two Indian cities, Ahmedabad and Gandhinagar, by using two Landsat 8 images (one for day and one for night) for each city. According to the findings, a model based on the relationship of LST with indices (NDVI, NDBI, ISF) improves the correlation of day and night LST. Similarly [23], used a Land Cover Contribution Index ((LCCI) to examine the contribution of each LULC cover to UHI formation (LCCI). The study's findings revealed that replacing vegetation with impervious surfaces has a significant impact on increasing UHI intensity [24]. used NDVI, Enhanced Vegetation Index (EVI), Normalized Difference Built-up Index (NDBI), Urban Index (UI), Normalized Difference Soil Index (NDSI), and Normalized Difference Water Index (NDWI) for downscaling LST of MODIS data with LULC indices and found that NDBI was the more suitable for downscaling LST using Distrad Model. Some researchers examined the relationship between LST, LULC, and NDVI in a mountainous region but in a small study area with two dimensions (North-South and West-East) [25]. The current study assessed LST, LULC types, and NDVI dynamics and their relationship with topographic elements in the Lower Himalayan region with 4 dimensions (North-South, West-East, North-West to South-East, and North-East to South-West). Due to the size of the study area, the area was divided into four dimensions as each dimension has a different climatic regime.

## 2 Materials and methods

### 2.1 Study area

**Fig. 1:**
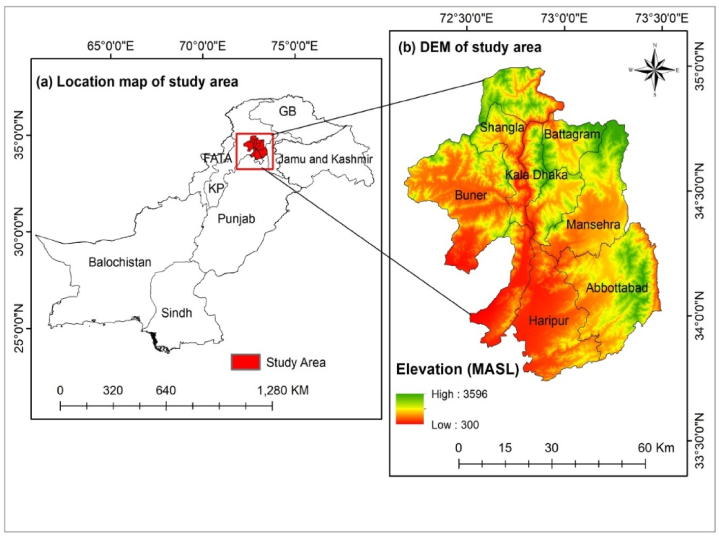
(a) Location map of the study area (Lower Himalayan region) within Pakistan, and (b) Elevation map showing various elevation bands and district boundaries of the study area.

The study area lies in the Lower Himalayas of Pakistan, consisting of the districts of Haripur, Buner, Abbottabad, Mansehra, and Shangala with geographical coordinates of 33°30′ to 35°30′ N Latitude and 72°–73°30′ E Longitude (Fig. 1a and b). It comprises an area of 9350 km^2^ with a population of 6 million people [26]. The maximum elevation ranges up to 3596 m above sea level in the study area (Fig. 1b). The climate varies from tropical at the base to snowy at higher elevations. The region is classed as humid subtropical climate (Cwa) at lower and medium elevations, and Subtropical highland climate (Cwb) at higher elevations, according to the Köppen climatic classification [27]. The monsoon is the most significant part of the climate of the study area, and most of the precipitation occurs in the spring. Local factors influence the climate and temperature declines by 6.5 °C for every 1000 m of altitude. This results in various climatic zones ranging from tropical in the foothills to permanent snow and ice at higher altitudes [28]. The windward slope receives more precipitation than the leeward slope. The lower southern Himalayan slope is less populated due to ‘Main Boundary Thrust.’ The northern slope is gentle, supporting pastures and terraced agricultural fields. Cereal-based agriculture fields exist at 2000 m above sea level, where densely inhabited areas along valleys and hills are located on the north side.

### 2.2 Data sets and methods

**Fig. 2:**
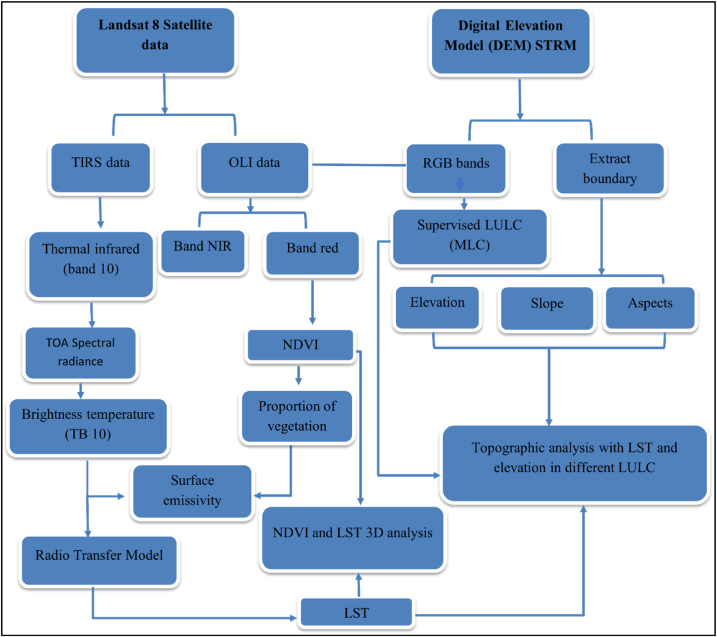
Methodology flow chart.

The data used in this study include Landsat 8 OLI/TIRS thermal infrared (band 10) remote sensing data, Digital Elevation Model (DEM), and Northern Pakistan shape file data. The Landsat 8 (OLI/TIRS) data was downloaded from the US Geological Survey (USGS) website (https://earthexplorer.usgs.gov). The Landsat 8 has two science instruments, Operational Land Imager (OLI) and Thermal Infrared Sensor (TIRS). The OLI sensors have the resolution of 30 and used to collect non thermal data such as land surface physical features while TIRS has the resolution of 100 m and used to collect LST data as shown in the (Fig. 2). The LST data was verified by using ground-based measurements. The cloud-free data from May 2021 was chosen to avoid cloud effects in the study area analysis. Overall downloaded Landsat 8 images has cloud cover of 9% and 16% for the Row-Path of 150/36 and 150/37, respectively but study area has no cloud cover. The details of the collected Landsat 8 data are given in Table 1. The collected Landsat 8 (OLI/TIRS) data is available on the USGS website in geometrically corrected form, and therefore, no geometric correction was applied. However, data were corrected for atmospheric distortions by Dark Object Subtraction (DOS) method in QGIS 2.8 software. The corrected Landsat data was then used for the generation of LULC, NDVI, and LST maps as shown in Fig. 2. The DEM file was obtained from the USGS website (https://earthexplorer.usgs.gov) and used for obtained slope, elevation and aspect of the study area. Pakistan shape file was downloaded from the Diva GIS website (https://www.diva-gis.org/gdata). After downloading Pakistan shape file, study area boundary was extracted in ArcMap 10.5 software. The relationship between LST, LULC, NDVI, and topographic elements was investigated by creating elevation-based profiles in (a) North-South (b) West-East (c) North-West to South-East (d) North-East to South-West direction with ArcMap 10.5 software. The slope file was used to evaluate LST in LULC classes with sunny and shady slopes at different elevations interval (100 m).

**Table 1:** Landsat satellite 8 images details used in the study (USGS).

Date	Scene ID	Cloud cover (%)	Sensor	Path/Row	Spatial resolution(m)
May 31, 2021	LC81500362021151LGN00 and LC81500372021151LGN00	9% and 16%	Landsat 8 OLI	150/36 and 37	15–30
			Landsat 8 TIRS	150/36 and 37	100

#### 2.2.1 Radiative transfer model

Remote sensing detects electromagnetic radiation (EMR) of various objects using sensors mounted on a remote sensing platform, allowing the spectral response from land surface characteristics to be analyzed [29]. The wavelength for LST measurements within the EMR spectrum is predominantly thermal infrared (TIR), ranging between 8 and 15 μm [1]. TIR-based sensors receive EMR in Top of Atmosphere (TOA) radiances [30]. During the daytime, there is both emission and reflection of EMR, but sensed EMR is restricted to only emission during the night. The inverse of Planck's law is used to derive blackbody/brightness temperatures from TOA radiances, which can be corrected and converted to LST [31]. [1], through different methods [32,33]. Vegetation indices such as NDVI measure changes in terrestrial vegetation because of their ability to absorb photosynthetically active radiation [21,34,35]. The NDVI data is obtained from different satellites like MODIS, Landsat, and AVHRR [36]. In the current study, the LST was estimated using a single-channel algorithm-based radiative transfer model [37,38]. The radiative transfer model is the base of LST inversion theory, which is used to estimate LST from remote sensing satellite data [39]. Several studies have shown that using radiative transfer model to estimate LST from satellite's remote sensing thermal data is more accurate than using universal single channel algorithm [40]. The current study used a radiative transfer model to estimate LST from Landsat 8 TIRS data. The LST estimation in the current study included satellite data pre-processing (radiation calibration), radiance calculation of TIRS band 10, atmospheric correction, NDVI and emissivity calculation, radiance calculation of black body at the same temperature, and estimation of LST [41]. The brightness temperature obtained from a remote sensing sensor is the amount of radiation expressed in terms of the temperature of a hypothetical blackbody emitting the same amount of radiation at the same wavelength. The inverse of the Planck function is used to calculate brightness temperature from radiation. Brightness temperature may be highly dependent or independent on the wavelength of the radiation depending on the properties of the source of radiation and any subsequent absorption. The brightness temperature must be converted and calibrated with surface emissivity. Emissivity is the ratio of the power emitted by a body at a known temperature to the power emitted if the body obeyed Planck's law of radiation. It is important to estimate LSE, as it reduces the errors during the estimation of LST from space. The following are the specific steps used for calculation of LST given in equation (1):

(1) Lsensor,i={(εiBi(Ts)+(1−εi)}Latm↓Ti+Latm↑

where Lsensor,i is the sensor's thermal infrared radiance for the ith band, and εi is the sensor's land surface specific emissivity for the i band (W/(m^2^ sr m)). Bi(Ts) represents the Planck black body thermal radiance (W/(m^2^ sr m)), where Ts is the LST in Kelvin (K). From the ground the total atmospheric transmittance to the sensor in band i is denoted by τi where Latm,i represents downward radiation and Latm,i represents upward radiation (W/(m^2^ sr m). The NASA's website (https://atmcorr.gsfc.nasa.gov/), were used for atmospheric parameters and Latm,i, and Latm,i were calculated using the imaging time, latitude, and longitude. The blackbody's thermal radiance Bi(Ts) was first determined using the thermal infrared band, and then Ts was estimated using the following Planck formula given in equation (2).

(2) $$Ts=\frac{K2}{K1\;Bi\;\left(Ts\right)+1}$$

where Bi(Ts) is the black body radiance value in the thermal infrared band while K1 and K2 were obtained from the image header file. The values of K1 and K2 were 774.89 W/m^2^ sr μm and 1321.08 W/m^2^ sr μm, respectively [32].

NDVI was used to calculate surface emissivity using the mixed image separation method [32,42]. The emissivity of most terrestrial materials lies between 0.7 and 1 [1], however, surfaces that have emissivity less than 0.85 are likely to be found in deserts. Unlike the emissivity of water bodies such as oceans, the emissivity of land surfaces may significantly differ from one place to another [30]. Emissivity may differ according to the viewing angle, surface moisture, and roughness as well as with vegetation. Although there are several methods available for emissivity calculation but due the presence of various LULC types in the study area, the mixed separation method was chosen [43]. The land surface was first divided into water, built-up areas, and natural surfaces. The water body had an emissivity of 0.995. The following equations (3), (4), (5) were used to calculate the surface emissivity values of built-up and natural surfaces:

(3) $$\varepsilon b=0.9588+0.0862Pv-0.0674{PV}^{2}$$

(4) $$\varepsilon s=0.9624+0.0620Pv-0.0462{PV}^{2}$$

where emissivity of built-up surface pixels and natural surface pixels are denoted by εb and εs, respectively and Pv is the vegetation area obtained by the following equation:

(5) $$Pv=\frac{NDVI-\left(NDVI\right)s}{\left(NDVI\right)v-\left(NDVI\right)s}$$

where NDVI stands for Normalized Difference Vegetation Index, (NDVI)v and (NDVI)s are the vegetation and bare soil NDVI values, respectively as shown in the (Fig. 2). In the current study Pv was approximated by setting (NDVI)v and (NDVI)s values to 0.7 and 0.05, respectively, as the pixels in the study area were not clearly covered by complete bare soil or vegetation. The value of Pv was 1 when the NDVI of a pixel exceeded 0.70, and 0 when it was less than 0.05; that is, when the NDVI of a pixel exceeded 0.70, the pixel was supposed to be totally covered by vegetation. The pixel was completely covered by bare soil when the value was less than 0.05. After estimating emissivity, LST was calculated using equations (1), (2)) from section 2.2.1.

#### 2.2.2 Normalize difference vegetation index

The differences in near-infrared and red reflectance of green vegetation were used to determine NDVI, which ranged from −1 to 1. The NDVI was calculated by the following formulae given in the (equation (6)) [32]:

(6) $$NDVI=\frac{\left(\rho 5-\rho 4\right)}{\left((\rho 5+\rho 4\right)}$$

where, ρ4 is the red band (0.64–0.67 μm) reflectance and ρ4 is the near-infrared band (0.85–0.88 μm) reflectance.

#### 2.2.3 Land use land cover types

The LULC maps were prepared using the Maximum Likelihood Classification (MLC) technique, based on the probability of the user-given training samples [44]. The MLC method chosen for LULC classification has some drawbacks, such as assigning higher likelihood to pixels, that leads to misclassification error. However, the total accuracy was above 80% which is the acceptable level of accuracy for LULC classification [8]. The study area LULC types were classified into built-up, bare soil, agriculture, vegetation, and water body. The accuracy of LULC classification was assessed using 40 ground truth points obtained from each LULC class during the field survey For LULC classification accuracy enhancement, ground control points, spectral and spatial profiles, and supplementary information from google earth images were used to develop training samples (Appendix 1 and Appendix 2). The confusion matrix method, which is extensively used for assessing the accuracy of LULC classification, was used to assess the accuracy of LULC classification as shown in appendix 3 [45].

#### 2.2.4 Sampling zone design

The Arc-Map 3D tool was used to assess the association between LST, elevation, and aspect. For this purpose, first LST and elevation raster maps were imported into ArcMap 10.5 software, and then the elevation profile was constructed using the 3D lines option of ArcMap 3D analysis. The study area profile was constructed in the following direction [13].

(a) North-South
(b) West-East
(c) North-West to South-East
(d) North-East to South-West

After constructing the elevation profile, random samples of LST and elevation were generated in Arc-Map 10.5 and for statistical correlation analysis IBM SPSS Statistics 25 software was used. The LST behaviour with LULC types at different elevation levels was also assessed along the constructed elevation profile. Zonal analyses were used to determine the mean LST and standard deviation in different LULC classes.

The LST in different LULC types was measured along sunny and shady slopes to investigate the effect of the sun angle in the study area. For this purpose, samples at 100 m intervals were collected for both sunny and shady slopes using DEM, slope, LST, and LULC (2021) maps. The spatial distribution of LST and NDVI with elevation and aspects was examined by creating combined LST and NDVI profile graph using the 3D analyst tool [13]. The relationship between NDVI and LST in different LULC types was also assessed using correlation analysis. For this purpose, first random samples were generated which were then analyzed in IBM SPSS software.

## 3 Results

### 3.1 Distribution of LST zones

**Fig. 3:**
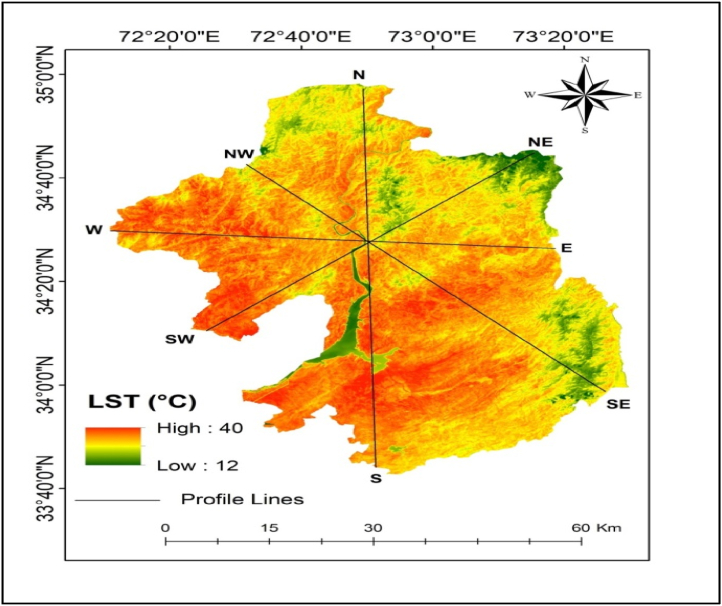
LST distribution map generated from Landsat 8 images from the year 2021 in the study area. Profile lines are superimposed on map using 3D analysis tool in ArcMap 10.5.

**Fig. 4:**
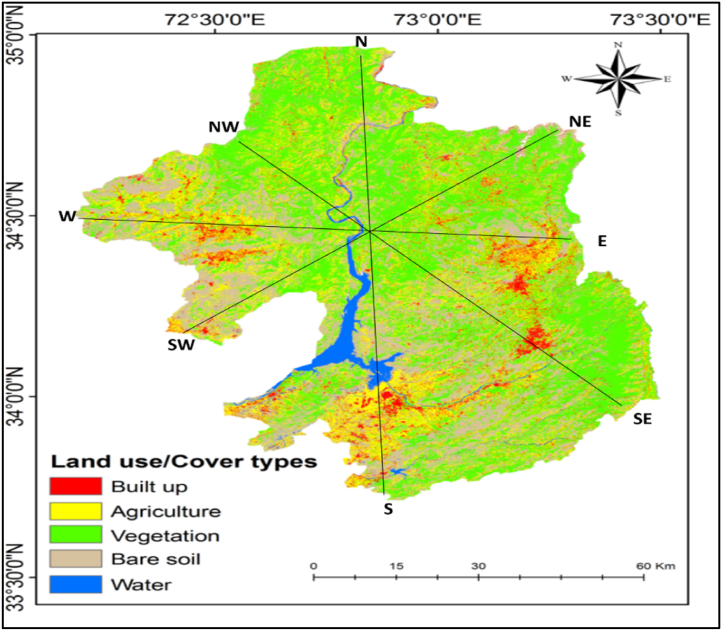
LULC types of maps of the study area generated from Landsat 8 images from the year 2021 in the study area.

**Fig. 5:**
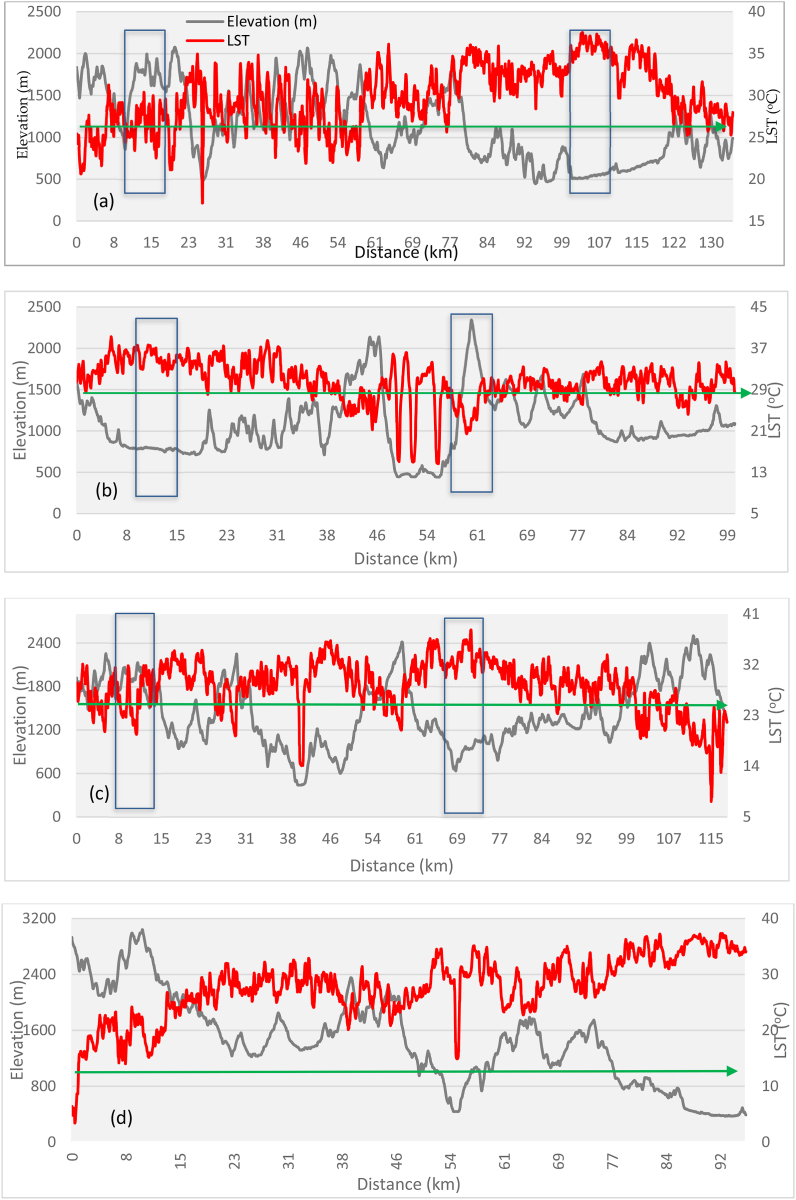
a, b, c, and d. LST and elevation profiles of the study area from North-South (a), West-East (b), North-West to South-East (c) and North-East to South-West (d).

The mean LST results for LULC types showed that the built-up areas have the highest mean LST (35 °C), followed by bare soil, agricultural land, vegetation, and water body (Table 2). High LST areas are mainly found in urban and suburban areas (Fig. 3, Fig. 4). The results indicated that low-elevation built-up areas have higher LST than high-elevation built-up areas, but high-elevation vegetation has lower LST. The LST in bare soil decreases as elevation increases (Fig. 1, Fig. 3, Fig. 4). The LST profile with elevation indicated an increasing trend in the northwest to southeast direction due to a decrease in elevation (Fig. 5a, b, c, and d). High (35 °C) and low LST (23 °C) peaks are found in the study area indicated by the blue mark boxes (Fig. 5a, b, c, and d). High LST peaks indicate urban areas, while low LST peaks show high-elevation areas of hills and valleys (Fig. 5).

**Table 2:** The mean LST of the various LULC types in the study area.

LULC Class	Built-up	Agriculture	Bare soil	Vegetation	Water body
Mean LST	35.76	26.18	28.09	23.49	18.25
Standard Deviation	2.37	0.55	2.12	1.70	1.22

### 3.2 Association between LST, elevation, and aspect

**Fig. 6:**
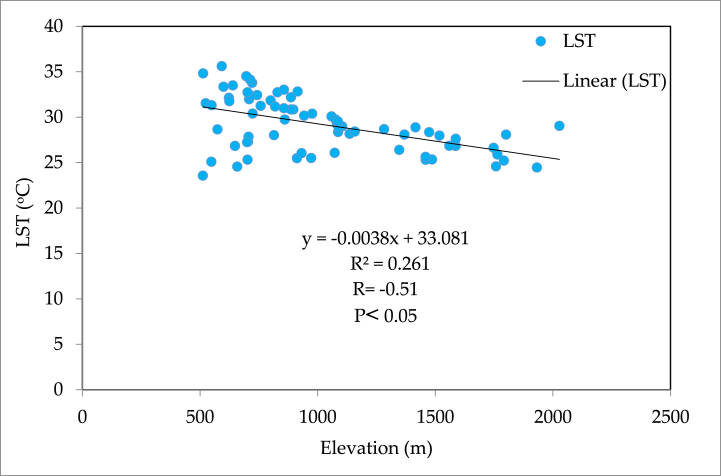
LST and Elevation correlation in the study area.

LST and elevation findings showed an inverse distribution pattern (Fig. 5), indicating that LST increases as elevation decreases. The LST is lower in high-elevation areas (the northern part of the study area) than in lower-elevation areas (the southern part of the study area). As we move south to north, LST steadily decreases with increasing elevation (Fig. 1, Fig. 5a, b, c, and d). Similar patterns have been observed when elevation increases in the west-east direction. The hills and valleys of the study area's north and west-eastern ends cause low and high LST peaks, depicted by blue boxes (Fig. 5a, b, c, and d). The correlation of LST with elevation was found negative with the R-value of −0.51 (Fig. 6). For plotting the graph, we used 70 random samples from elevation and LST files in ArcMap 10.5 software.

### 3.3 Association between LST, LULC, elevation, and slope direction

#### 3.3.1 The mean LST in various LULC types

The LST results for various LULC classes showed that built-up areas and bare soil have higher LST and standard deviation than water bodies, vegetation, and agricultural land (Table 2). The rate of increase or decrease in LST depends on the heat capacity of the material of each land cover type. For example, built-up areas in our study area had a high LST due to low heat capacity than other LULC classes. The bare soil and agricultural land had high heat capacity than built-up areas, therefore low LST as compared to the built-up area. The lowest LST was found in vegetation and water body, respectively.

#### 3.3.2 LST variations among LULC types based on slope direction and elevation

The elevation of the study area was from 600 to 2300 m above sea level (m asl). The results of the LST of different LULC types based on slope direction and elevation indicated that the mean LST of the sunny slope was higher than the shady slope (0.80). This difference was calculated from mean slope wise LST variation in all LULC classes between sunny and shady slope (Table 3). The LST was ranked from higher to lower as built-up > bare soil > agricultural > vegetation > water on both slopes. Meanwhile, LST also showed variation with elevation among the LULC classes (Table 3). The correlation coefficient between elevation and LST was negative, with an R-value of −0.51 (Fig. 6).

**Table 3:** LST of the various LULC types on sunny and shady slopes in different elevation bands in the study area.

Elevation (m ASL)	LST (ᵒC) on sunny slopes in various LULC types					LST (ᵒC) on shady slope in various LULC types				
	Built-up	Agriculture	Vegetation	Bare soil	Water	Built-up	Agriculture	Vegetation	Bare soil	Water
600–700	**33.25**	**31.80**	**29.90**	**32.4**	**26.90**	**33.01**	**30.93**	**28.80**	**32.2**	**26.85**
700–800	32.83	31.22	28.55	32.45	26.73	32.6	30.70	27.83	31.3	26.50
800–900	32.77	29.94	28.40	32.11	26.63	32.55	29.2	27.55	31.22	26.43
900–1000	32.15	29.11	-	32.87	26.62	32.36	29.01	-	31.05	26.21
1000–1100	31.8	28.9	-	-	26.32	31.96	28.7	-	-	26.06
1100–1200	31.23	27.42	27.31	31.91	26.14	31.22	27.2	27.2	31.88	26.12
1200–1300	30.11	27.33	27.25	28.9	25.34	30.02	27.15	27.01	28.77	25.26
1300–1400	30.95	26.8	26.95	27.77	24.71	30.70	26.45	26.11	27.6	24.45
1400–1500	**29.75**	**25.99**	26.02	**26.7**	24.63	29.7	**25.85**	25.70	**26.45**	**24.33**
1500–1600	-	-	25.11	-	24.63	**28.45**	-	25.45	-	23.63
1600–1700	-	-	24.55	-	23.86	-	-	24.45	-	23.86
1700–1800	-	-	24.30	-	19.98	-	-	24.08	-	19.72
1800–1900	-	-	23.94	-	19.88	-	-	23.11	-	19.88
1900–2000	-	-	22.50	-	19.55	-	-	22.19	-	18.95
2000–2100	-	-	21.87	-	**19.22**	-	-	20.93	-	**18.02**
2100–2200	-	-	**21.79**	-	-	-	-	19.18	-	-
2200–2300	-	-	-	-	-	-	-	**18.22**	-	-
LULC & elevation-wise LST variation	+3.50	+5.81	+8.11	+5.7	+7.68	+4.56	+5.08	+10.58	+5.75	+8.83
Mean Slope-wise LST variation	6.16					6.96				
Total slope-wise LST difference	**0.80**									

The vegetation class had the highest LST variation, with values of (+8.11) and (+10.58) respectively, on the sunny and shady slopes, while the lowest variation was in the built-up class on both sides (Table 3). The highest variations among all the classes were at the elevation of 600–700 m asl, while the lowest variation in most of the classes (3 out of 5) was at the elevation of 1400–1500 m asl (Table 3).

### 3.4 Association between LST, NDVI, and aspect

#### 3.4.1 Spatial distribution of LST and NDVI

**Fig. 7:**
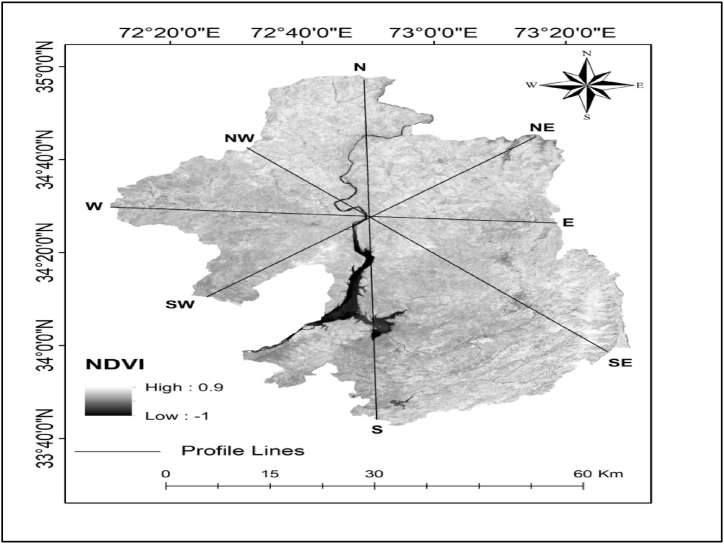
NDVI spatial distribution of the study area.

**Fig. 8:**
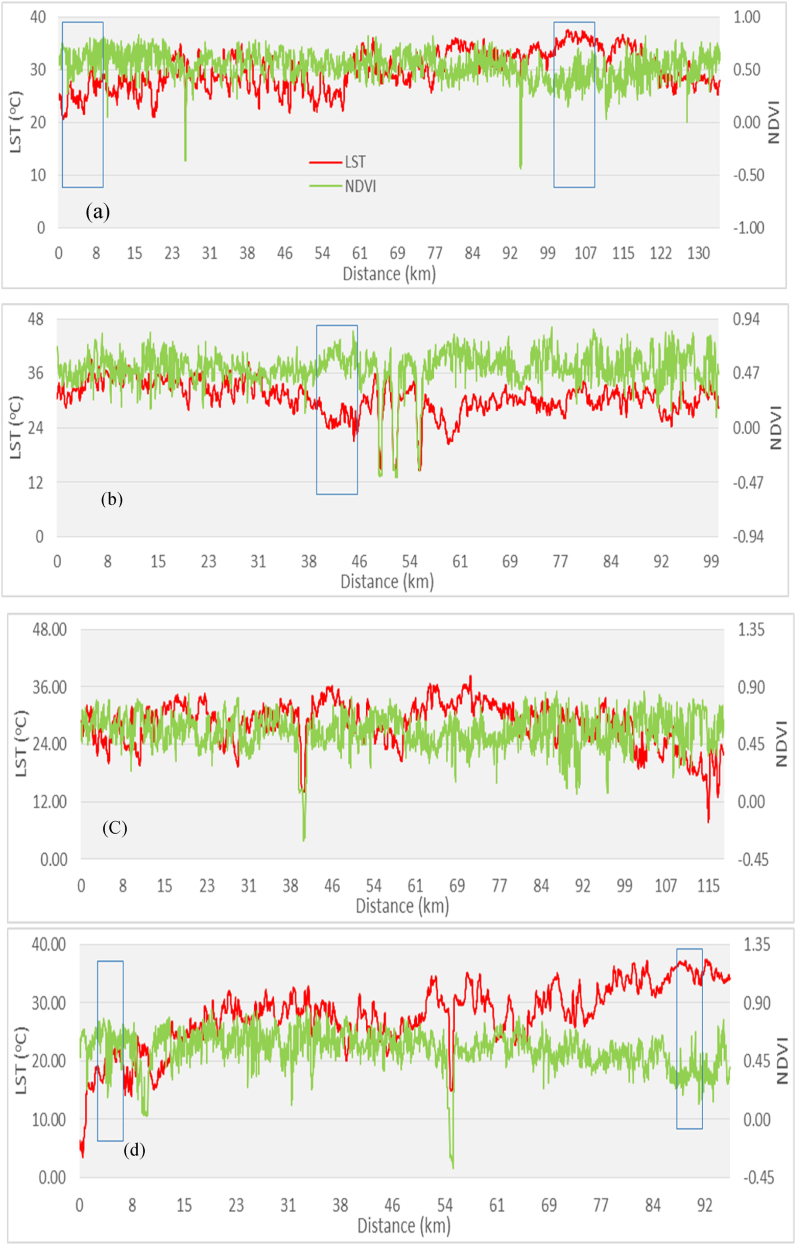
a, b, c, and d. NDVI and LST profiles of the study area from North-South (a), West-East (b), North-West to South-East (c), and North-East to South-West (d).

**Fig. 9:**
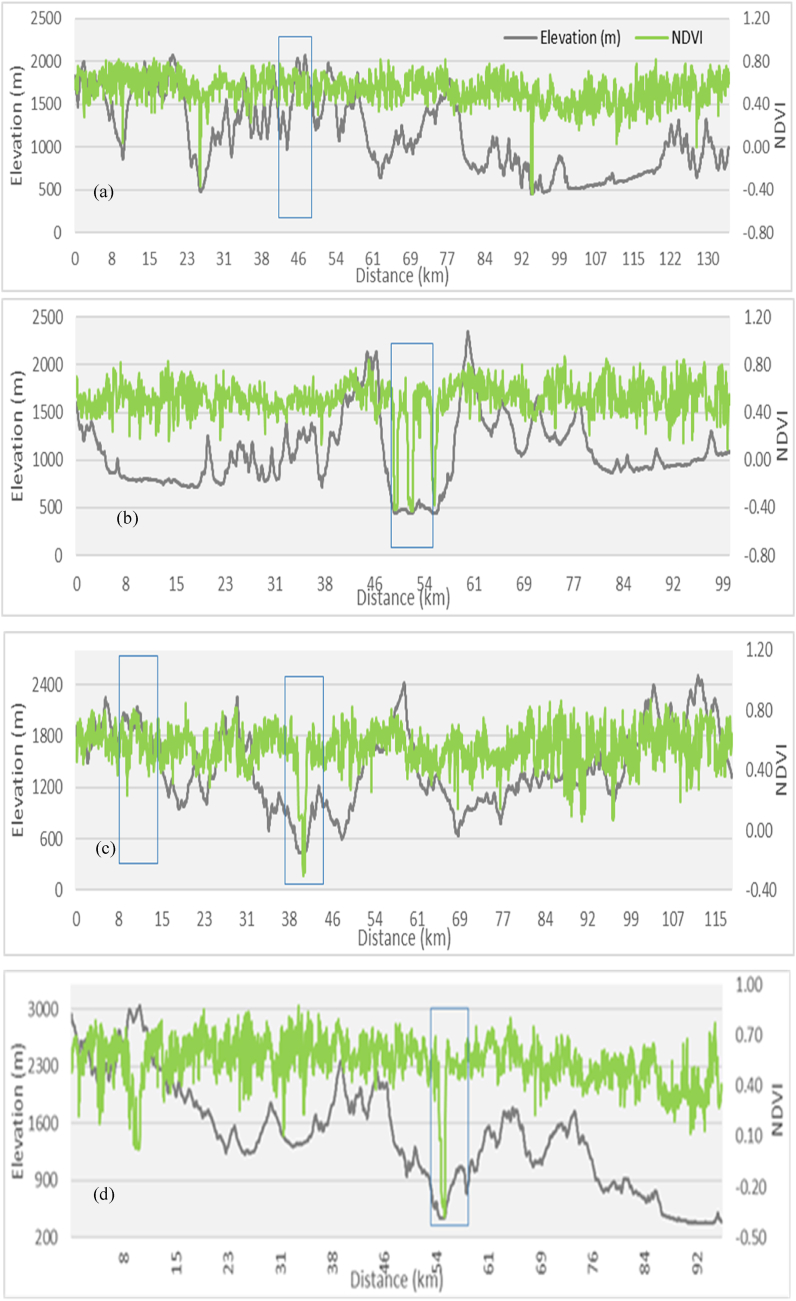
a, b, c, and d. NDVI and Elevation profiles of study area from North-South (a), West-East (b), North-West to South-East (c) and North-East to South-West (d).

LST and NDVI findings revealed an opposing spatial distribution pattern (Fig. 8a, b, c, and d), i.e., the NDVI value was low (0.5) in the lower elevation southern portion of the study region and high (0.8) in the higher elevation northern part, but high LST was found in the southern part (0.4) and low LST in the northern part (Fig. 6, Fig. 7). The lowest NDVI was found in the water body which was 0.2. Similarly, high LST was observed in built-up areas (35.76 °C) and bare soil (28.09 °C), whereas lower LST (24.49 °C) was noticed in the vegetation with high NDVI values (Table 2 and Fig. 9a, b, c, and d). The water body LST remained relatively stable (18.25 °C) with zero vegetation coverage (Fig. 8a, b, c, and d). showed rectangular boxes with high NDVI values in the study area. High and low LST and NDVI peaks are found in the study area indicated by the blue mark boxes (Fig. 8a, b, c, and d). High NDVI peaks indicate low LST vegetative areas, while low NDVI peaks show high LST urban areas. Similarly (Fig. 9 a, b, c, and d) blue boxes indicated high NDVI values in vegetative areas located at higher elevations of the study area.

#### 3.4.2 Quantification of association among LST and NDVI in various LULC types

LST has a negative relationship with NDVI in all LULC classes except for the water body (Table 4). The highest negative correlation coefficient was shown by built-up areas, followed by bare soil, agriculture, vegetation, and water body (Table 4). Waterbody sampling data has been removed in order to determine whether any difference was caused by the water or not. LST and NDVI showed a negative relationship after water was removed. The results showed that P-Value was less than .00001. The outcome, as shown in Fig. 6, was significant at p < .05.

**Table 4:** Correlations between LST and NDVI and regression equations were generated based on these relationships in different LULC of the study area.

LULC types	Correlation (NDVI-LST)	R^2^	Regression Equation
Built up	−0.517	0.267	Y = 29.465+ (−2.833)*NDVI
Agriculture	−0.254	0.064	Y = 38.202+ (−8.464)*NDVI
Vegetation	−0.629	0.395	Y = 36.788+ (−10.928)*NDVI
Bare soil	−0.376	0.141	Y = 35.643+ (−8.399)*NDVI

## 4 Discussion

### 4.1 High land surface temperature zones

The spatial distribution of LST had been significantly impacted by built-up areas in the studied region. However, in addition to high LST zones caused by built-up areas, other high LST zones were also found in low elevation non-built-up areas, most likely caused by bare soil areas since the area primarily consists of exposed rocks and non-vegetative sub-urban areas. Over the previous decades, rapid urbanization and deforestation in the study area resulted in a loss of vegetation, exposing bare soil and rock to sunlight in the study area [46]. This decreases thermal inertia and surface heat energy leading to high LST zones [44]. In addition to urbanization and deforestation, uneven topography also led to high LST zones. For example, the northern part of the study area has hills and valleys, which result in high LST. In summary, the LST of Northern Pakistan was featured not only by conventional UHI effects but also by high LST zones due to deforestation, LULC types, and topography. Similar findings have been reported by Ref. [47], from Jaipur, India, and [13], in the Karst region of China.

### 4.2 Association between LST, elevation, and aspect

The study region's elevation increased from south to north and southeast to northwest while the LST decreased, indicating that elevation regulates its role in LST. A similar trend was reported in the southeast direction. The southern portion of the study areas has the highest LST while the northern portion has the lowest LST. This is due to the fact the southern portion has lower elevation as well as is dominated by urban areas. This LST pattern is due to two major factors, namely (1) LST is affected by air temperature, which is lower at higher elevations, and (2) the good surface vegetation in mountainous regions of the study area [48]. Similar effects of elevation on LST have been reported by Ref. [49], from the western Sichuan Plateau of China and [50], in Northwest Vietnam. Our study findings also revealed LST differences between slope directions due to differences in solar radiation intensity and length between slope directions (Table 3), which is consistent with [51]. For example, the mean LST difference between sunny and shady slopes was 1.02 C° in LULC classes. In this study, at the same elevation in the same slope direction, the LST of the different LULC types showed variations. This difference in LST in different LULC types is attributed to surface roughness, thermal conductivity, thermal capacity, and surface albedo [52].

### 4.3 Association between LST, LULC types, elevation, and slope direction

Our results of LST among different LULC types (Table 2) were consistent with [6], who found that impervious surfaces have higher LST than non-impervious surfaces, and with the assessment of [53], who mentioned the effect of LULC changes on LST in an urban environment. LST in the study area is affected by several factors, such as the cooling effect of evapotranspiration, surface roughness, albedo, and solar radiation. For example, vegetation and water body has lower LST than built-up areas due to the cooling effect of evapotranspiration. [54], found that LST varied by 1 °C with slope and aspect due to the difference in solar radiation received by LULC types. LST is also affected by surface roughness and albedo, which can be seen because the built-up areas have less roughness and low albedo than vegetation, therefore having high LST (Table 2). The effect of slope direction on the mean LST was different because the solar radiation received by the earth varies in different slope directions, which led to the difference in the LST [13,55]. The LST also varies with elevation and LULC types, which were also found by Ref. [56], while assessing the effect of slope direction and elevation on LST in southern Italy.

### 4.4 Association between LST and NDVI and aspects

LST and NDVI were influenced by underlying surface types and elevation due to the cooling effect of the vegetation surface [57], caused by the evapotranspiration cycle. Built-up and bare soil has low NDVI and high LST due to impervious surfaces. The NDVI and LST for the various LULC types showed a negative statistical relationship, except water body, which remained relatively stable, and showed similarity with findings of [7,58]. The high negative relationship between NDVI and LST (Table 4) is due to seasonal variations, as found by Ref. [7], and [59]. Similarly [60], reported a negative correlation between LST and NDVI for the summer season, while the winter season has a positive correlation for the same year. Since our study area image is from the summer season, which may be the main reason for the negative correlation between LST and NDVI in different Land classes. The relationship of LST with NDVI was still negatively correlated with removing water body data, which is limited to the findings of other researchers [61,62].

## 5 Conclusions

The radiative transfer model was used for the estimation of LST from Landsat 8 data (TIRS) in this study. The LST spatial distribution pattern and influencing factors such as elevation and slope-aspect in Northern Pakistan were studied by using elevation and LULC types of data. The LST was then evaluated in various LULC types, and the spatial distribution and quantitative relationship of NDVI and LST in the study area were discussed. Our study's main findings were as follows: (1) The LST was influenced by topography and LULC type. The LST in the study area decreased from south to north. (2) The statistical correlation between LST and elevation was negative for the study area. (3) The LST results for different LULC types showed that the built-up area has the highest mean LST (35.76 °C), while water (15.25 °C) has the lowest LST. (4) Water body has low NDVI and LST. (5) The statistical correlation between NDVI and LST was found to be negative in the quantitative analysis. (6) The water body had a positive correlation with LST. (7) The LST difference between the sunny and shady slopes was 1.02 °C. In conclusion. The LST of the study area is controlled by topographic elements and underlying surface types. These study findings could be used for LULC planning as well as ecological and environmental restoration in Pakistan's lower Himalayan region.

## Author contribution statement

Waheed Ullah; Khalid Ahmad; Siddique Ullah; Adnan Ahmad Tahir; Muhammad Faisal Javed; Abdul Nazir; Arshad Mehmood Abbasi; Mubashir Aziz; Abdullah Mohamed: Conceived and designed the experiments; Performed the experiments; Analyzed and interpreted the data; Contributed reagents, materials, analysis tools or data; Wrote the paper.

## Funding statement

This work was supported by King Fahd University of Petroleum and Minerals.

## Data availability statement

Data will be made available on request.

## Declaration of interest’s statement

The authors declare no conflict of interest.
