# Supplementary material for: Analysis of the relationship among land surface temperature (LST), land use land cover (LULC), and normalized difference vegetation index (NDVI) with topographic elements in the lower Himalayan region
Source: Heliyon. 2023 Feb 3;9(2):e13322. doi: 10.1016/j.heliyon.2023.e13322 (PMC9942242; doi:10.1016/j.heliyon.2023.e13322)
Supplement: Multimedia component 1 [file mmc1.docx]

Appendix 1

Table (a) Ground control points for accuracy assessment

| S. No | Latitude | Longitude |
| --- | --- | --- |
| 1 | 34.195444 | 73.220059 |
| 2 | 34.193476 | 73.210395 |
| 3 | 34.857693 | 72.654556 |
| 4 | 35.017555 | 72.653183 |
| 5 | 34.470451 | 72.256508 |
| 6 | 34.310663 | 72.675362 |
| 7 | 34.349142 | 73.185900 |
| 8 | 34.34807 | 73.186705 |
| 9 | 34.348291 | 73.189934 |
| 10 | 34.346830 | 73.189516 |
| 11 | 34.813664 | 72.739219 |
| 12 | 34.816388 | 72.739894 |
| 13 | 34.174191 | 73.236768 |
| 14 | 34.199926 | 73.250895 |
| 15 | 33.986887 | 72.880566 |
| 16 | 34.024064 | 72.925798 |
| 17 | 33.991584 | 72.909748 |
| 18 | 33.998273 | 72.932922 |
| 19 | 34.174777 | 73.220487 |
| 20 | 34.193522 | 73.240057 |
| 21 | 34.328170 | 73.193263 |
| 22 | 34.306903 | 73.207683 |
| 23 | 34.671136 | 73.018387 |
| 24 | 34.676183 | 73.028086 |
| 25 | 34.497007 | 72.836852 |
| 26 | 34.519640 | 72.766814 |
| 27 | 34.149109 | 73.312240 |
| 28 | 34.137043 | 73.322569 |
| 29 | 34.144560 | 72.827422 |
| 30 | 34.320544 | 72.859007 |
| 31 | 34.749847 | 72.817989 |
| 32 | 34.754149 | 72.823911 |
| 33 | 34.825007 | 72.750343 |
| 34 | 34.838111 | 72.739185 |
| 35 | 34.672824 | 73.025433 |
| 36 | 34.673013 | 73.026484 |
| 37 | 34.211408 | 73.263366 |
| 38 | 34.180389 | 73.271912 |
| 39 | 33.988168 | 72.870438 |
| 40 | 33.974076 | 72.981331 |


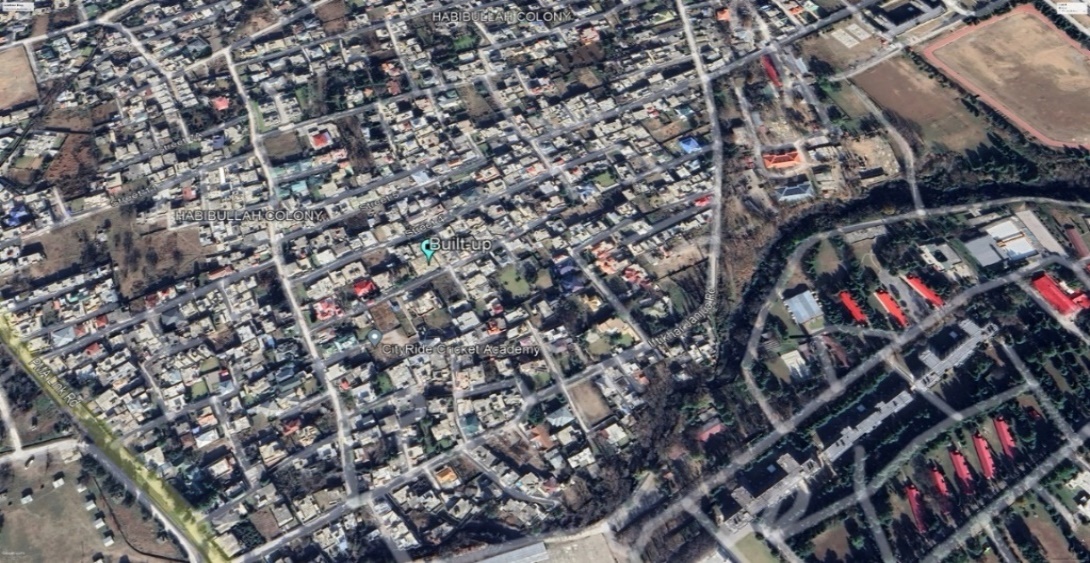
Appendix 2


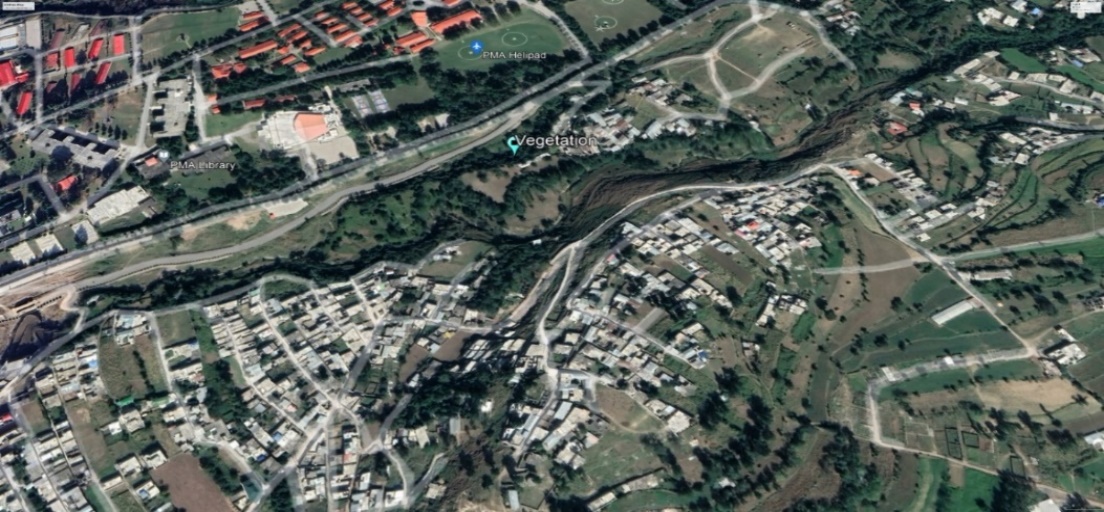
Fig. (a) Google earth image of built-up features


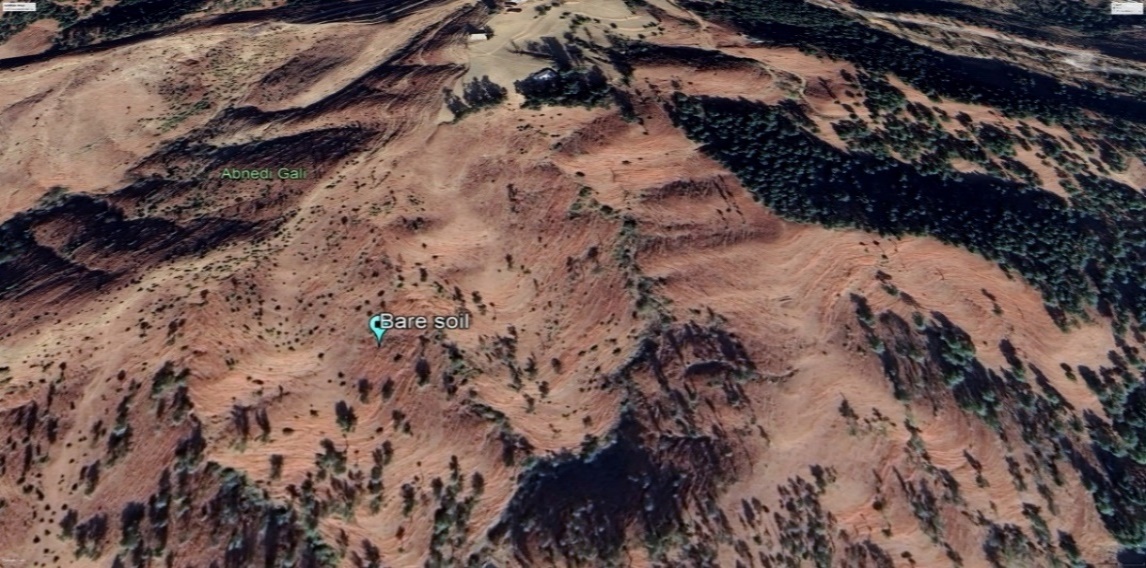
Fig. (b) Google earth image of vegetation features

Fig. (c) Google earth image of bare soil features


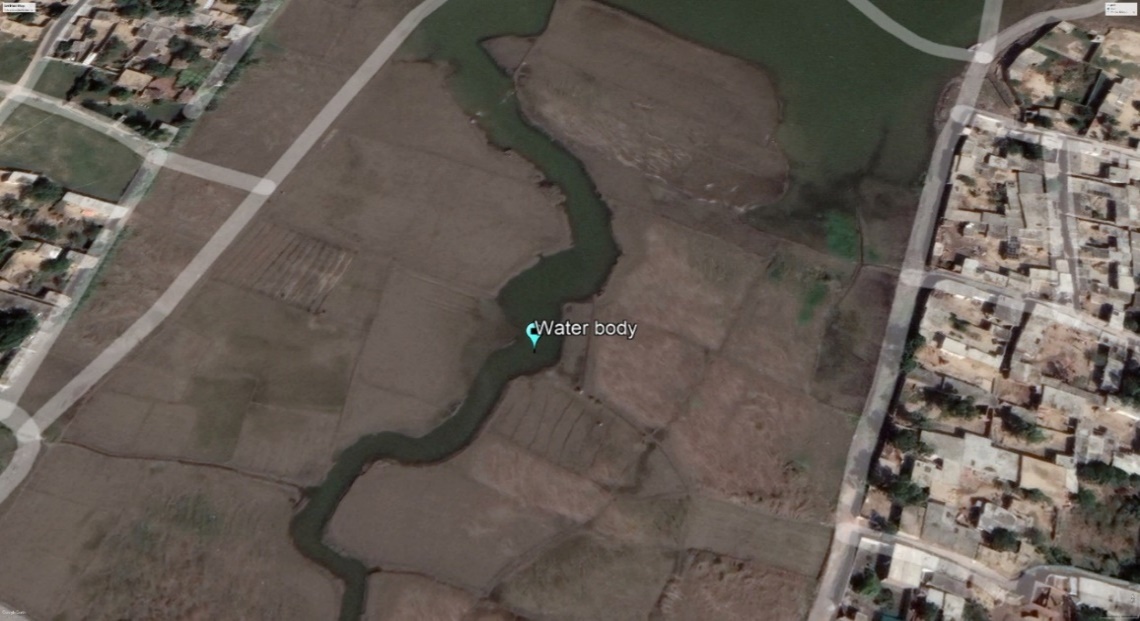

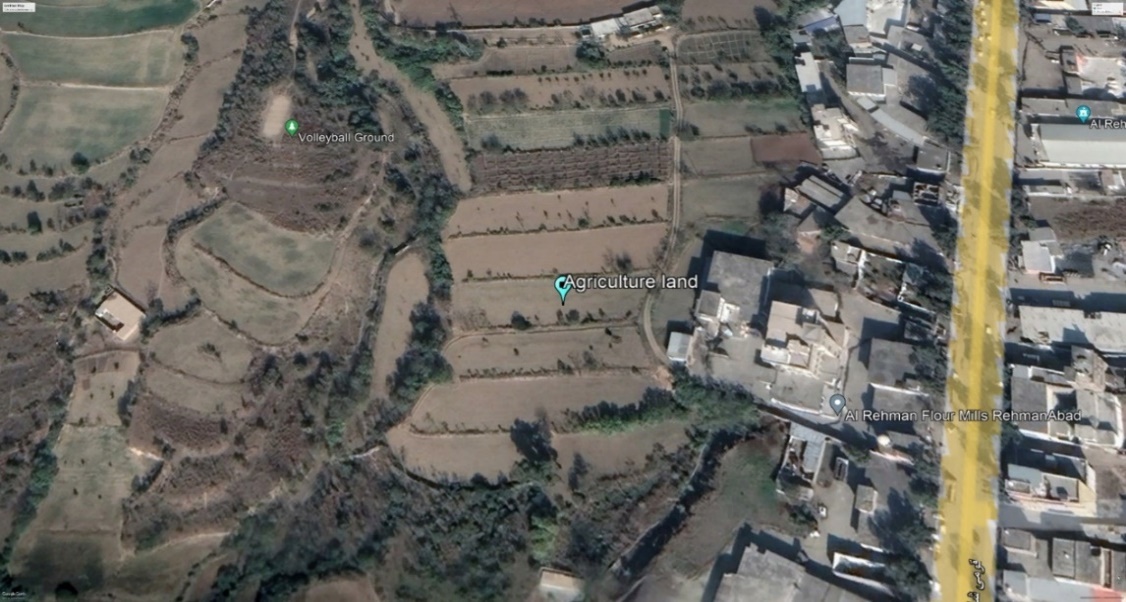
Fig. (d) Google earth image of agriculture land features

Fig. (e) Google earth image of water body features

Appendix 3

Table (b) Accuracy assessment of the land use/cover types image for the year 2021

| Year | Users  Accuracy (%) | Producers  Accuracy (%) | Overall  Accuracy (%) | Kappa  coefficients |
| --- | --- | --- | --- | --- |
| 2021 | 89.11 | 88.80 | 89.91 | 0.86 |
